# Precarity and clinical determinants of healthcare-seeking behaviour and antibiotic use in rural Laos and Thailand

**DOI:** 10.1136/bmjgh-2020-003779

**Published:** 2020-12-09

**Authors:** Marco J Haenssgen, Nutcha Charoenboon, Thipphaphone Xayavong, Thomas Althaus

**Affiliations:** 1 Global Sustainable Development, University of Warwick, Coventry, West Midlands, UK; 2 Institute of Advanced Study, University of Warwick, Coventry, West Midlands, UK; 3 Population Health Sciences, Bristol Medical School, University of Bristol, Bristol, UK; 4 Jacobs, Cordova & Associates, Vientiane, Vientiane Capital, Lao People's Democratic Republic; 5 Centre for Tropical Medicine and Global Health, University of Oxford Centre for Tropical Medicine, Oxford, UK; 6 Mathematical and Economic Modelling, Mahidol Oxford Tropical Medicine Research Unit, Bangkok, Thailand

**Keywords:** health policy, public health, community-based survey, infections, diseases, disorders, injuries

## Abstract

**Background:**

The social determinants of health are a decisive yet persistently understudied area for tackling global health challenges like antimicrobial resistance (AMR). Precarity is one determinant whose importance is increasingly recognised, which we define here as ‘a form of pernicious self-dependence that undermines individuals’ control over their own lives and limits their ability to flexibly respond to crises’. We aimed to assess the relationship between precarity, other forms of deprivation and healthcare-seeking behaviour by asking, ‘What is the impact of precarity, marginalisation and clinical presentation on healthcare-seeking behaviour?’ and ‘Do patients experiencing precarious livelihoods have clinically less advisable healthcare-seeking behaviour?’

**Methods:**

We used healthcare-seeking behaviour census survey data from rural Thailand and Laos, wherein five rural communities were surveyed two times over a period of 3 months (2-month recall period). Using descriptive statistical and multivariate logistic regression analysis on the illness level, we studied precarity alongside clinical presentation, marginalisation and facilitating solutions during an illness (eg, health-related phone use) as determinants of healthcare-seeking behaviour in the form of healthcare access and antibiotic use.

**Results:**

The data included 1421 illness episodes from 2066 villagers. Patients in precarious circumstances were up to 44.9 percentage points more likely to misuse antibiotics in the presence of situational facilitators (predicted antibiotic misuse: 6.2% (95% CI: 0.9% to 11.4%) vs 51.1% (95% CI: 16.6% to 85.5%) for precarious circumstances with/without facilitation). Marginalisation was linked to lower antibiotic use, but this did not translate into clinically more advisable behaviour. Clinical presentation played only a minor role in determining healthcare access and antibiotic use.

**Conclusions:**

This study underlines the importance of context and local livelihoods in tackling drug resistance. While supporting the growing emphasis on AMR-sensitive development policy, we call for future research to study systematically the healthcare-seeking behaviour impact of precarious livelihoods, social policy and community development initiatives.

**Trial registration number:**

NCT03241316.

**Key questions**

**What is already known?**

- Precarity is an important social determinant of health with demonstrated physical and mental health consequences, which affects people in low-income, middle-income and high-income contexts.
- The response to antimicrobial resistance (AMR) in human use focuses heavily on individual behaviour change, neglecting the critical role that contextual determinants play in behaviours of individuals.
- The behavioural impact of precarious livelihoods is hypothesised to contribute to clinically inadvisable antibiotic use and therefore potentially to AMR.

**What are the new findings?**

- We used an original micro-level behavioural data set from Thailand and Lao People’s Democratic Republic, which enabled us to disentangle clinical presentation, marginalisation and precarity as separate drivers of healthcare-seeking behaviour.
- The link between clinical presentation and antibiotic use was surprisingly weak. Instead, patients in precarious circumstances were significantly more likely to misuse antibiotics in the presence of situational facilitators (eg, mobile phones and social support activated during an illness).
- This is the first study that examined quantitatively the relationship between precarity and AMR-related healthcare-seeking behaviour.

**Key questions**

**What do the new findings imply?**

- Development processes that change whether and how people experience precarious circumstances could have unforeseen implications for collective global health threats such as AMR.
- Our study lends support to ‘AMR-sensitive development policy’: global health interventions must move beyond patient-centric and disease-centric approaches, acknowledging and responding to contextual factors that shape how people cope with illness.
- If precarity as a social determinant continues to be neglected, then localised forms of hardship could unwittingly influence and undermine the effectiveness of existing clinical and behavioural interventions to tackle AMR.

## Background

Global health research and practice have become increasingly sensitive to non-clinical factors that influence health outcomes. This recognition has been driven by the concepts of the ‘social determinants of health’ and ‘diseases of poverty’,^1–3^ which draw attention to the role of the local context in the response to global health priorities. Antimicrobial resistance (AMR) is one of these priorities, feared to become one of the leading global causes of death by 2050^4^—and we here study the role of precarity as a contextual factor shaping patients’ healthcare-seeking behaviour with reference to AMR (we define precarity as ‘a form of pernicious self-dependence that undermines individuals’ control over their own lives and limits their ability to flexibly respond to crises’—that is, a condition driven by contextual factors that deprive people of predictability and stability of their lives; see further explanation below).

One key domain of demand-side responses to AMR is population behaviour change towards a more targeted use of antimicrobials such as antibiotics (in line with clinical presentation).^5^ With education and awareness campaigns being key policy instruments,^5 6^ the behaviour change approach in AMR assumes that the main drivers of behaviour are patient misconceptions and a lack of knowledge.^7–10^ However, the link between antibiotic use and people’s ‘awareness’ of drug resistance (and their level of education more generally) is more complicated than is often assumed in AMR policy documents.^11–15^ In addition, an approach that foregrounds awareness and education also implicitly prioritises individual over contextual factors of healthcare-seeking behaviour—such as precarity.

In a growing and interdisciplinary body of literature, precarity has been receiving attention as a social determinant of health,^16 17^ linked especially to employment and working conditions.^3 18 19^ Driven by stress and economic insecurity, it is argued that work-related precarity contributes to physical and mental illness and, ultimately, to premature mortality.^1 3 17^ Outside of public health, Butler^20^ describes precarity as the unfair distribution of social, economic and political structures that could protect people from ‘disease, poverty, starvation, displacement and (…) exposure to violence’.^20^ Examples of precarity-inducing circumstances defined thus include oppressive gender norms and experiences of ‘disempowerment’.^21 22^

Patients in such circumstances could find themselves pressured into detrimental patterns of self-medication and delayed healthcare access. Consequently, ‘quick fix’ solutions such as self-medication or antibiotic purchase without prescriptions can be interpreted as default strategies to cope with adversity,^23–25^ or perhaps even as ‘performance enhancers’.^26^ Precarity could therefore challenge the underlying assumptions and ultimately the effectiveness of public awareness campaigns that form the backbone of global AMR strategies (‘Only take antibiotics prescribed to you’).^4 8 27^ However, and despite the growing body of work, evidence evaluating contextual factors leading to precarity against the backdrop of AMR is limited (empirical research has studied the broader link between precarity and health but with a tendency to focus on high-income contexts^28–30^). In response, we investigated two research questions against the backdrop of AMR: ‘What is the impact of precarity, marginalisation and clinical presentation on healthcare-seeking behaviour?’ We subsequently explored whether these patterns of healthcare-seeking behaviour are justified in light of patients’ clinical presentation: ‘Do patients experiencing precarious livelihoods have clinically less advisable healthcare-seeking behaviour?’

We situated our research in rural Thailand and Lao People’s Democratic Republic (PDR), considering that Southeast Asia is an epicentre for antibiotic resistance.^31–34^ Rural settings are of particular relevance because their health systems face heightened infrastructural, human resource, and financial challenges and a high burden of communicable diseases.^35–38^ Precarity in various forms has been highlighted in the region as well,^39 40^ whereby large parts of working populations remain excluded from social welfare schemes; informal agricultural work alternates with off-season casual labour (eg, in factories) and gradual environmental degradation and the expanded presence of industry and international companies exacerbate precarious livelihoods.^41–44^ These circumstances have been associated with potentially detrimental forms of healthcare-seeking behaviour, such as unsupervised self-medication.^45^

## Methods

### Study sites and population

Compared with Lao PDR, Thailand exhibited a more advanced economic and health system context, evidenced by virtually zero extreme monetary poverty at US$1.90/day (Lao PDR: 23%), government healthcare expenditure of 15% of total government expenditure (Lao PDR: 4%) and an expected life expectancy at birth of 75 years (Lao PDR: 67 years) (2017 data from World Bank^46^). Thailand also had a more established AMR strategy, which was evident in a national action plan on AMR together with a network of 74 surveillance sites—neither of which existed in Lao PDR at the time of this research.^34^

Our study population was the general adult population of rural Chiang Rai province, Thailand, and Salavan province, Lao PDR. The specific study sites comprised five villages (three in Chiang Rai, two in Salavan), which were selected in consultation with local stakeholders. Guiding selection criteria were (1) village size and structure, (2) remoteness, (3) village-level infrastructure, (4) ethnic composition and (5) available health facilities. The villages had an estimated population between 339 and 1462 residents (including children; estimates based on enumeration roster data), representing 3331 villagers in total (1125 in Chiang Rai, Thailand; 2206 in Salavan, Lao PDR).

### Patient and public involvement

This project was based on preceding qualitative research with local northern Thai patients and the general public,^47 48^ which revealed the need for sociomedical research on patient experiences of illness in context. In this particular project, members of the Thai and Lao public were involved through cognitive interviewing to inform the survey design and through local workshops to improve our understanding of local medicine uses and healthcare-seeking behaviour.

### Survey design and implementation

We implemented two-round individual-level census surveys in the five purposively selected villages between November 2017 and May 2018. Within the selected communities, all households were enumerated using a satellite-aided sampling approach,^49^ and all adults (aged at least 18 years) were invited to participate. Our household definition was based on a shared kitchen and previous residence of at least 6 months in the survey village. The household response rate in the first survey round was 97.9%, and 99.1% in the second survey round (745 and 754 out of 761 households, respectively). A total of 2066 adult villagers were interviewed, whereby 88.8% (1678/1890) of the first-round participants could be re-interviewed (taking into consideration non-response and seasonal migration).

We chose an oral consent procedure to not alienate and discriminate against illiterate or unregistered participants. Of the 3744 responses across the two survey rounds, we did not receive consent for data sharing in four instances, which constitutes the main difference between the data analysed here and the publicly available data set of our project.^50^ The recruitment and data collection processes were monitored in the field through the survey supervisors, a research officer and digital survey monitoring tools. 15.0% (560/3744) of all interview sessions were monitored by a survey supervisor.

Based on the complete village census, we identified as patients those respondents who had an acute illness and injury in the 2 months preceding the survey (or children below 18 years who experienced an illness episode under the respondents’ supervision).

### Survey instrument

Our survey instrument was a 45-min face-to-face questionnaire (see online supplemental file 2), administered on tablet computers using SurveyCTO.^51^ The locally recruited field teams received 5 days of full-time classroom and field training (plus an additional 5 days for supervisors). The questionnaire was piloted in rural Chiang Rai and Salavan, with 50 cognitive interviews supporting the questionnaire development and revision as well as the contextualisation of the survey data (not reported here).^52^ The questionnaire was co-developed with the local research team in English, Thai and Lao, administered in the local language (Thai/Lao), and translation difficulties with ethnic minority languages were resolved by recruiting local translators within the survey villages.

10.1136/bmjgh-2020-003779.supp2 Supplementary data

The questionnaire modules included (1) basic demographic and socioeconomic information, (2) social network structures, (3) antibiotic-related knowledge and attitudes and (4) healthcare-seeking behaviour during acute illnesses and accident-related injuries. This study used in particular modules 1 and 4, whereby 60.8% (1256/2066) of the participants volunteered information about at least one recent illness episode, experienced by themselves or a child under their supervision within the 2 months preceding the survey, yielding a total of 1887 illness episodes captured by the community census surveys (1421 episodes after removing ongoing/yet unresolved illnesses).

The healthcare-seeking data included self-reported symptoms and recalled diagnosis of the condition as well as detailed information about each step of the illness episode, from the moment a discomfort was detected until the condition was resolved or the symptoms otherwise disappeared. For every step, the type of treatment was recorded (including ‘ignoring’ the condition, self-care and care from family and friends, and various locally specific forms of informal and formal treatment) together with the type of received medication (as recalled by respondent) and supplementary information about the process (eg, mode of transport to reach point of care). This information formed the basis for our analysis of healthcare-seeking behaviour: on the one hand, these data documented ‘revealed’ healthcare-seeking behaviour during the course of an illness episode (we focused here specifically on whether formal healthcare access took place and on the medicine that patients received during the illness episodes). On the other hand, the rich information on self-described symptoms and recalled diagnoses (which we discussed and interpreted together with the local research teams) permitted a broad categorisation of clinical presentation (see online supplemental table 1) and—together with information on disease severity, duration and recurrence alongside the revealed healthcare-seeking behaviour of the patient—enabled a basic clinical evaluation by our medically qualified research team member (TA; described in more detail below).

10.1136/bmjgh-2020-003779.supp1 Supplementary data

Considering the importance of antibiotics for our research question, we focused on cases where we could establish antibiotic use with a high degree of confidence. We manually coded free-text responses of reported medicines and treatments based on pharmaceuticals in local circulation, and triangulated ambiguous responses (eg, ‘germ killer’) based on respondents’ reports as to how they colloquially refer to antibiotics in module 3 of the survey questionnaire. Of all 4611 recorded medicine use episodes, 13.5% (623/4611) involved an antibiotic with a high degree of confidence (8.2% of all illness episodes did not involve any form of treatment, while 23.3% could not be classified as either antibiotic or non-antibiotic—eg, ‘white powder’ or ‘green capsule’—given the available information).

### Determinants of healthcare-seeking behaviour

The key independent variables comprise clinical determinants and composite indexes of precarity, marginalisation and situational facilitation. We explain these variables below and provide variable summaries in online supplemental table 2.

#### Clinical determinants

The impact of each clinical determinants, their duration, severity (mild, moderate and severe) and frequency over the past 6 months was evaluated on healthcare-seeking behaviour. The nine clinical determinants were based on respondents’ reported symptoms and recalled diagnosis during medical encounters, and included presence of an infection, fever, respiratory tract symptoms, common cold (defined as the presence of runny nose and/or sneezing and/or cough), digestive symptoms, uro-gynaecological symptoms, neurological symptoms, traumatism and other symptoms. For full disclosure, the complete list of symptoms and their coding into clinical presentation is provided in online supplemental table 1.

#### Precarity

The health impact of precarity is often framed in similar terms as problematic employment conditions, poverty/marginalisation (which are often used synonymously) or stress.^1 3 17 53 54^ Based on our definition, however, we argue that precarity is a phenomenon with distinct behavioural implications. Precarity differs from poverty and marginalisation (which are often used as synonyms in the context of multidimensional deprivation), because it can be experienced regardless of wealth level (in middle-income and high-income contexts as well as among populations that are not conventionally deemed ‘poor’),^55–59^ and it may have different impacts on healthcare-seeking behaviour than the ‘barriers’ created by marginalisation (eg, living far away from a healthcare unit^54 60^). In relation to stress (which often leads precarity to be represented by employment conditions), it is important to note that the behavioural influence of precarity only partly overlaps with stress experienced in problematic employment or under conditions of poverty.^61 62^ More broadly, the behavioural impact of precarity may be better described as a ‘loss of control and flexibility’ over health-related decisions, which extends beyond employment and also involves for instance community-level social support.^21 25 63^

In order to represent the distinct circumstances under which precarity could militate against healthcare-seeking behaviour, we considered six indicators along occupational, social and logistical dimensions. The occupational dimension clearly cannot be ignored in a study of precarity and evaluated whether (1) people’s income was erratic (eg, no formal employment contract or regular income) and whether (2) the structure of the work restricted people’s flexibility when responding to illnesses (daily labourers and agriculturalists).^25 30^ The social dimension of precarity was evaluated through the (3) absence of other adults in the households and (4) the lack of a health-related social network (in terms of regular exchange of health advice^38 64 65^). Lastly, logistical indicators comprised the absence of solutions to flexibly address health problems, namely (5) no household mobile phone (as consistent predictor of individual non-use of mobile phones during an illness^66^) and (6) no motor transport option within the household (including motorcycles/tricycles, cars or tractors).

#### Marginalisation

Marginalisation was evaluated in terms of education, wealth and ethnicity^60 67^: (1) the respondent had not received any formal education, (2) belonged to the lowest household asset quintile among the rural population in their province and (3) did not belong to the majority Thai/Lao Loum ethnic groups in their field sites (who might also speak different languages). Although spatial marginalisation would also be an important dimension to consider, we chose not to include it into the index considering that distances to health facilities were similar and clustered at the village level. The respondents’ distance to the nearest formal health facility was thus included as a separate control variable,^38 67^ alongside other controls including the sex of the respondent and whether the reported illness episode was experienced by the respondent themselves or a child (below 18 years of age) under their supervision.^21 67^

#### Facilitation

In addition to the indicators above, situations and practical means may arise situationally—that is, revealed during the course of an illness—which enable patients to respond to crisis and escape the structural conditions of precarity and marginalisation.^21 68^ Our survey detected such ‘situational facilitators’ in the form of tools and support sources that become visible during an illness episode: (1) support received from other people during the illness episode, (2) health-related phone use during the illness (not necessarily by the patient themselves) and (3) the use of a car or taxi during the illness (eg, from a neighbour). These situational facilitators could help mitigate structural conditions of precarity, as a result of which we analyse them separately and in interaction with our precarity index.

### Behavioural outcomes

Outcome variables considered in this study comprised different forms of access to healthcare and antibiotic use to represent healthcare-seeking behaviour. Reflecting the pluralistic health systems of Southeast Asia,^69^ healthcare access (outcome variable ‘Access’) included formal healthcare providers (public primary care units, hospitals, private clinics) and informal sources of care (pharmacies, unlicensed private practitioners, traditional healers, grocery stores, family members and friends). Antibiotic use (outcome variable ‘Antibiotic’) captured whether any antibiotic was received at any point during the illness episode (including antibiotics stored at home).

We complemented the analysis of healthcare-seeking behaviour with an evaluation of clinically ‘inadvisable’ antibiotic use and healthcare access. These evaluations used the comprehensive survey data surrounding patient clinical presentation (symptoms, symptom severity, duration, recurrence), which enabled us to identify cases in which healthcare access and antibiotic use appeared clinically advisable but was not pursued (or vice versa). Given the nature of the self-reported data, we did not flag behaviours as ‘inadvisable’ where the clinical indication was unclear. The evaluation was carried out by a medically qualified co-author with research experience in the field sites (TA) in correspondence with the local research teams.

Specifically, the first clinical indicator (outcome variable ‘Inadvisable_Access’) evaluated the rationale for seeking no formal treatment despite a clear indication for care, and vice versa. The second clinical indicator evaluated the rationale for antibiotic use (outcome variable ‘Inadvisable_Antibiotic’), whereby our focus was on identifying situations with a clear indication to not use an antibiotic, namely scrub typhus, leptospirosis, cholera and malaria. We also flagged as inadvisable where patients used antibiotics despite the absence of a clear indication for an antibiotic (namely common cold, documented viral or fungal or parasitic illness, chronic pain and bone trauma) and when patients accessed antibiotics stored at home, from friends, traditional healers and other unregulated sources of healthcare (6% or 83/1421 illness episodes; robustness checks focusing only on clinical indication reproduced the results and were omitted from reporting.)

Note that these evaluations are strictly from a clinical perspective and do not entail judging villagers’ behaviour as ‘unreasonable’—our argument rather is that people have good reasons and structural determinants for behaving the way they do, and that this behaviour might look clinically inappropriate but requires non-clinical responses.

### Analytical strategy

The analysis took place on the illness level, whereby we analysed the pooled sample of all completed illness episodes across the two survey rounds. We proceeded in four steps:

- Step 1: descriptive analysis of the healthcare-seeking behaviour, precarity and marginalisation among the study population.
- Step 2: contribution of the clinical determinants on healthcare-seeking behaviour.
- Step 3: contribution of precarity and marginalisation on healthcare-seeking behaviour.
- Step 4: the role of precarity as contributor to clinically inadvisable healthcare access and antibiotic use.

Step 1 was based on descriptive statistical analysis to describe and contrast the survey results across the two field sites. We compared the samples on the illness level, using χ^2^ tests for binary and Wilcoxon rank-sum tests (two-sided) for non-normally distributed variables to test for differences across sites.^70 71^

We conducted univariate and multivariate regression analyses for Steps 2–4 (see online supplemental file 1 for a description of the statistical models). For Step 2, we focused on the contribution of the clinical presentation to the healthcare-seeking outcomes (access to healthcare, antibiotic use). For each outcome, we separately estimated the contribution of the eight clinical presentations (excluding the heterogeneous group of ‘other symptoms’), controlling for symptom duration, severity and illness frequency as well as other confounding variables (see full variable list in online supplemental table 2). Step 3 assessed the relative contribution of precarity, facilitation and marginalisation to the outcomes of healthcare access and antibiotic use (excluding specific symptoms for simplicity but retaining other clinical determinants as control variables). In Step 4, we analysed whether precarity in isolation or moderated by situational facilitators contributes to healthcare-seeking behaviour that appears clinically less desirable (‘Inadvisable_Antibiotic’ and ‘Inadvisable_Access’), again controlling for clinical determinants and other control variables.

For all analyses in Steps 2–4, we first conducted univariate analyses, followed by multivariate analyses adjusted by confounders (the main results only contain multivariate results for simplicity). We initially chose multilevel logistic regression models with a site random effect.^72^ However, in several instances, the maximum likelihood estimation of the multilevel models failed to converge and to produce SE estimates,^73^ as a result of which we limited the presentation of the main results to single-level models with a site dummy.

We also conducted the multivariate analysis with an interaction effect between precarity index and facilitation index to consider the potential moderating effect of the situational facilitators. We compared model fitness between the interacting and non-interacting models based on the significance levels of the parameter estimate for the interaction term and the Akaike Information Criterion (AIC^74^), reporting interacting models where the interaction term was statistically significant (p<0.05) and AIC was smaller than the non-interacted model.

Our robustness checks (not reported here) included comparisons between the single-level and multilevel models and estimations using nested models and including patients’ symptoms as additional determinants of health behaviour. The robustness checks did not substantively influence the main results nor the conclusions of this paper.

We used the statistical software Stata V.15 (StataCorp). Throughout the results, we indicated significance levels below 0.1, 0.05 and 0.01 with *, ** and ***, respectively, and considered the 5% level as ‘statistically significant’.

## Results

### Step 1: patterns of illness, precarity and marginalisation

Across the 1421 illness episodes in the sample (625 in Chiang Rai and 796 in Salavan), the most commonly reported illness was the presence of any infection with 64.6% (918/1421) of all cases, while the term ‘fever’ was cited in 25.7% (365/1421) of all cases. Respiratory tract symptoms were the second most reported complaint (65.1%, 925/1421 cases), of which common cold represented 56% (519/925 cases). Median duration of symptoms was 5 days (IQR 3–8), with a moderate severity. 79.2% (1125/1421) of illnesses involved access to healthcare and antibiotic intake was declared in 28.7% of all cases (408/1421).

The results of the descriptive statistical comparison of the two field sites are presented in table 1. The comparison indicated that illness episodes were statistically significantly more likely to involve healthcare access and antibiotic use in Salavan, where healthcare-seeking behaviour was also more commonly misaligned with patients’ clinical presentation. The most common forms of clinical presentation in both sites were the presence of an infection, followed by respiratory tract symptoms (with common cold as the most frequent component) and the declaration of fever. Furthermore, precarity and marginalisation were more concentrated among the patients in Chiang Rai, as indicated by the relatively higher average precarity scores (0.39 vs 0.35, p<0.01) and marginalisation scores (0.56 vs 0.06, p<0.01). While not necessarily representative of the overall provincial population,^15^ these statistics reflect the living conditions of the specific field site communities.

**Table 1:** Illness, precarity and marginalisation across the field sites

Variable name	Chiang Rai n=625		Salavan n=796		Difference (χ^2^/z-scores)*	Variable description
	Mean	95% CI	Mean	95% CI		
Outcome variables						
Access	66%	0.62 to 0.69	90%	0.88 to 0.92	121.66***	Binary variable: patient accessed any kind of informal/formal care (1=yes)
Antibiotic	15%	0.13 to 0.18	39%	0.36 to 0.43	97.18***	Binary variable: antibiotic use during illness (1=yes)
Inadvisable_Access	28%	0.25 to 0.32	38%	0.35 to 0.42	14.73***	Binary variable: formal healthcare access without indication or vice versa
Inadvisable_Antibiotic	11%	0.09 to 0.14	22%	0.19 to 0.25	29.69***	Binary variable: antibiotic use without indication or vice versa, or antibiotic use from informal sources
Clinical presentation						
Sepsis	57%	0.53 to 0.61	71%	0.67 to 0.74	28.50***	Binary variable: presence of an infection
Respi	56%	0.52 to 0.59	73%	0.70 to 0.76	45.02***	Binary variable: respiratory presentation (incl. common cold)
Common_Cold	34%	0.31 to 0.38	38%	0.35 to 0.42	2.17	Binary variable: common cold
Fever	20%	0.17 to 0.23	30%	0.27 to 0.33	16.83***	Binary variable: fever
Neuro	12%	0.09 to 0.14	24%	0.21 to 0.27	33.49***	Binary variable: neurological presentation
Digest	16%	0.14 to 0.19	14%	0.12 to 0.16	1.58	Binary variable: digestive presentation
Uro_Gyneco	1%	0.00 to 0.02	2%	0.01 to 0.03	0.80	Binary variable: uro-gynaecological presentation
Trauma_Pain	16%	0.13 to 0.18	11%	0.08 to 0.13	7.77***	Binary variable: traumatism
Other	07%	0.05 to 0.09	3%	0.02 to 0.04	10.97***	Binary variable: other symptoms
Duration_Symptoms	1.48	1.44 to 1.53	1.36	1.32 to 1.39	4.02***	Categorical variable: duration of symptoms (1 =<7 days; 2=7–30 days; 3=>30 days)
Severity	1.79	1.73 to 1.85	1.82	1.77 to 1.87	−1.26	Categorical variable: symptom severity grade (1=low; 2=moderate; 3=severe)
Frequency	49%	0.45 to 0.53	35%	0.31 to 0.38	30.31***	Binary variable: repeated illness of patient within 6 months (1=yes)
Other independent variables						
Precarity_index	0.39	0.38 to 0.41	0.34	0.33 to 0.35	5.86***	Discrete variable: precarity index (composed of six individual indicators)
Marginalisation_index	0.56	0.53 to 0.59	0.06	0.05 to 0.07	26.69***	Discrete variable: marginalisation index (composed of three individual indicators)
Facilitation_index	0.38	0.36 to 0.41	0.26	0.24 to 0.28	7.48***	Discrete variable: facilitation index (composed of three individual indicators)
Control_adult	20%	0.17 to 0.23	38%	0.35 to 0.41	55.68***	Binary variable: illness of adult or child (0=adult, 1=<18 years)
Control_sex	57%	0.54 to 0.61	64%	0.60 to 0.67	5.75**	Binary variable: sex of respondent (1=female)
Control_distance	6.00	5.91 to 6.09	0.49	0.46 to 0.52	32.40***	Continuous variable: distance to nearest formal healthcare provider (km)

Illness-level data, including only completed illnesses experienced by respondent or a child under their supervision. Unweighted statistics.

Source: authors, based on survey data.

Χ^2^ test scores for binary variables and z-scores for Wilcoxon rank-sum tests for other non-normally distributed variables (Duration_Symptoms, Severity, Precarity_index, Marginalisation_index, Facilitation_index and Control_distance).

*p<0.10, **p<0.05, ***p<0.01.

### Step 2: clinical determinants and healthcare-seeking behaviour

**Figure 1:**
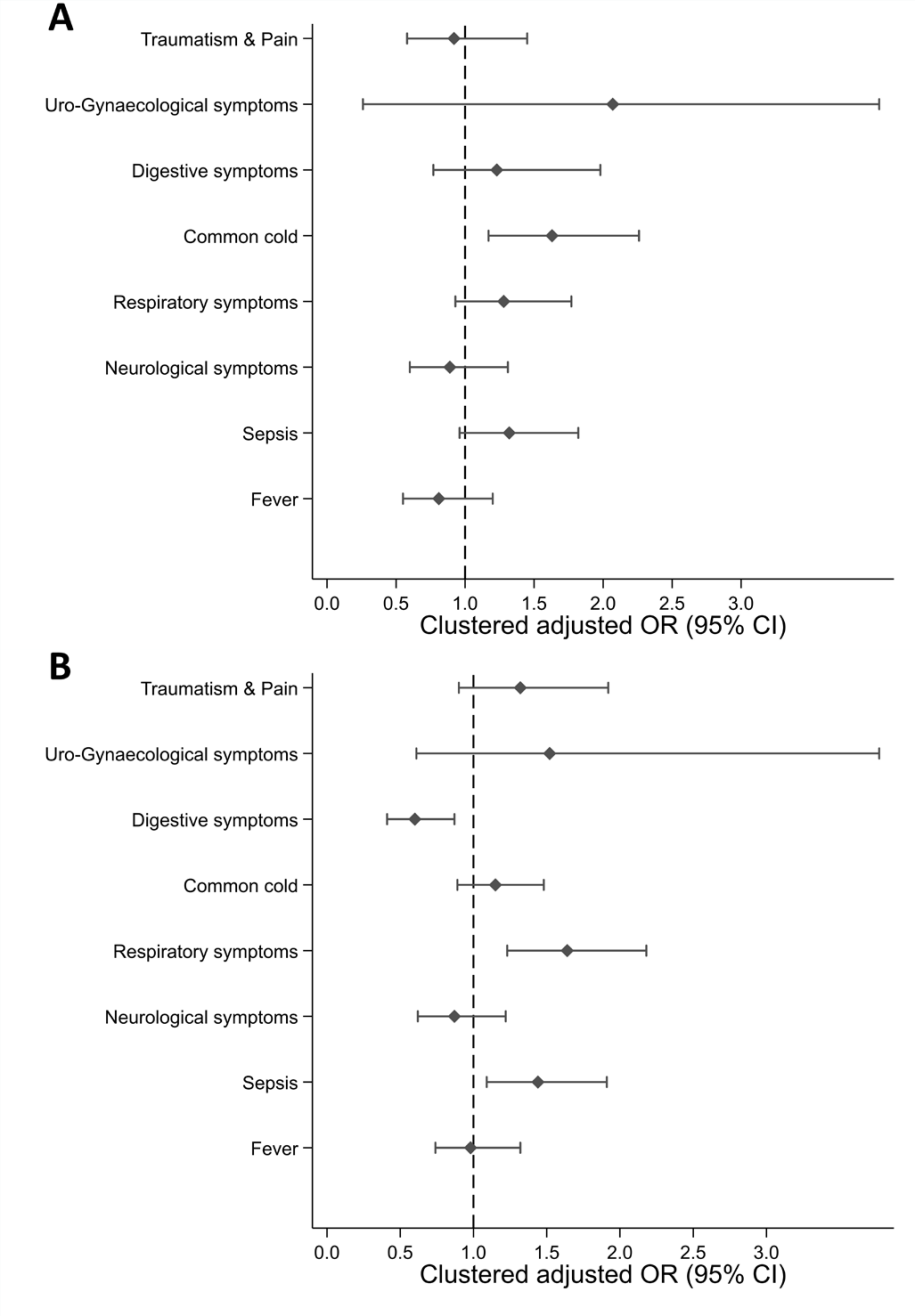
(A) Access to healthcare and (B) antibiotic intake according to clinical determinants in Chiang Rai and Salavan. Source: authors, based on survey data. Notes: OR adjusted by precarity, marginalisation, facilitation, duration, frequency and severity of symptoms, gender, age category, distance to the nearest formal healthcare and by cluster, using a site-fixed control variable. Error bars indicate 95% CI.

In the multivariate analysis of clinical determinants, the common cold was the only clinical presentation significantly associated with healthcare access (83.2% (432/519) of all common cold episodes; adjusted OR (aOR) 1.63 (95% CI: 1.17 to 2.26)). However, antibiotic intake for common cold was not significantly greater than other clinical presentations (aOR 1.15, 95% CI: 0.89 to 1.48), whereas the presence of an infection was significantly associated with antibiotic intake (32.5%, 298/918, aOR 1.44, 95% CI: 1.09 to 1.91), as was respiratory tract infection (33.1%, 306/925, aOR 1.64, 95% CI: 1.23 to 2.18). The presence of digestive symptoms was significantly associated with antibiotic withholding (22.8%, 49/215, aOR 0.60, 95% CI: 0.41 to 0.87). The association between clinical determinants and outcomes is illustrated in figure 1, with detailed results provided in online supplemental tables 3 and 4.

### Step 3: marginalisation and precarity as determinants of healthcare-seeking behaviour

Table 2 presents the results of the regression analysis, controlling in all cases for a reduced set of clinical determinants (duration, frequency and severity of symptoms) and considering the potential moderating role of situational facilitators in the interaction models (models 2 and 4).

**Table 2:** Determinants of healthcare-seeking behaviour

Dependent variable	Access (any access to healthcare)		Antibiotic (antibiotic use during illness)	
Model type	No interaction	Interaction model	No interaction	Interaction model
Model number	(1)	(2)	(3)	(4)
Marginalisation_index	1.02 (0.52 to 1.97)	1.03 (0.53 to 2.00)	0.31*** (0.16 to 0.60)	0.31*** (0.16 to 0.60)
Precarity_index	2.37* (0.92 to 6.10)	3.88** (1.11 to 13.58)	0.71 (0.33 to 1.56)	0.36* (0.11 to 1.21)
Facilitation_index	17.49*** (8.95 to 34.18)	40.50*** (8.39 to 195.59)	1.95*** (1.21 to 3.14)	0.96 (0.33 to 2.79)
Precarity_index # Facilitation_index		0.11 (0.00 to 4.43)		7.71 (0.49 to 120.87)
Control_adult (ref: adult)	4.60*** (2.86 to 7.41)	4.64*** (2.88 to 7.48)	1.57*** (1.20 to 2.05)	1.58*** (1.21 to 2.06)
Control_sex (ref: male)	0.97 (0.71 to 1.33)	0.97 (0.71 to 1.32)	1.25* (0.96 to 1.63)	1.26* (0.97 to 1.65)
Control_distance	0.94 (0.78 to 1.14)	0.94 (0.77 to 1.14)	0.93 (0.77 to 1.12)	0.93 (0.77 to 1.12)
Duration_Symptoms (ref: <7 days)				
7–30 days	2.40*** (1.66 to 3.46)	2.42*** (1.67 to 3.50)	1.72*** (1.31 to 2.25)	1.71*** (1.31 to 2.24)
*>*30 days	0.97 (0.43 to 2.18)	0.96 (0.43 to 2.17)	0.72 (0.35 to 1.51)	0.73 (0.35 to 1.53)
Frequency (ref: no repeat illness)	1.04 (0.76 to 1.42)	1.03 (0.75 to 1.40)	1.05 (0.81 to 1.35)	1.05 (0.81 to 1.36)
Severity (ref: low)				
Moderate	2.00*** (1.43 to 2.78)	1.98*** (1.42 to 2.75)	1.26 (0.95 to 1.68)	1.28* (0.96 to 1.70)
Severe	3.84*** (2.28 to 6.49)	3.82*** (2.26 to 6.46)	1.61*** (1.12 to 2.32)	1.63*** (1.13 to 2.34)
Site (ref: Chiang Rai)	4.94*** (1.84 to 13.27)	4.99*** (1.86 to 13.42)	1.52 (0.59 to 3.89)	1.53 (0.60 to 3.93)
Constant	0.01*** (0.00 to 0.10)	0.01*** (0.00 to 0.09)	0.09** (0.01 to 0.68)	0.11** (0.01 to 0.84)
Observations	1421	1421	1421	1421
P value	<0.01	<0.01	<0.01	<0.01
AIC	1075.27	1075.91	1542.65	1542.53

Source: authors, based on survey data.

Illness-level data, including only completed illnesses experienced by respondent or a child under their supervision. Unweighted statistics. ORs adjusted by all covariates. 95% CIs in brackets.

*p<0.10, **p<0.05, ***p<0.01.

AIC, Akaike Information Criterion.

The interaction terms were not statistically significant for healthcare access and antibiotic use. Instead, facilitation presented a strong and statistically significant positive association with healthcare access (aOR 17.49, 95% CI: 8.95 to 34.18) and antibiotic use (aOR 1.95, 95% CI: 1.21 to 3.14) independently of precarity. The marginalisation index was associated with antibiotic withholding at the 1% level (aOR 0.31, 95% CI: 0.16 to 0.60), whereas the precarity index was not statistically significantly associated with healthcare access or antibiotic use. Among other control variables, illnesses involving children, those with a higher severity rating and illnesses with a duration between 7 and 30 days were significantly more likely to involve healthcare access and antibiotic use.

### Step 4: precarity and clinically inadvisable healthcare-seeking behaviour

This final step assessed the factors contributing to clinically advisable healthcare-seeking behaviour (table 3). The interaction term was statistically significant at the five-percent level for the antibiotic use model. No statistically significant association existed between marginalisation and the dependent variables, whereas a significant association existed between precarity and ‘Inadvisable_Antibiotic’ through the interaction term. The statistically significant interaction term suggests that the association between precarity and inadvisable antibiotic use increased with higher levels of facilitation. Facilitation was also associated with more advisable patterns of healthcare access (model 1, aOR 0.33, 95% CI: 0.21 to 0.52).

**Table 3:** Determinants of clinically inadvisable healthcare access and antibiotic use

Dependent variable	Inadvisable_Access (formal healthcare access without indication or vice versa)		Inadvisable_Antibiotic (antibiotic use without indication or vice versa, or antibiotic use from informal sources)	
Model type	No interaction	Interaction model	No interaction	Interaction model
Model number	(1)	(2)	(3)	(4)
Marginalisation_index	1.04 (0.60 to 1.78)	1.04 (0.61 to 1.79)	0.61 (0.30 to 1.26)	0.61 (0.29 to 1.26)
Precarity_index	1.88* (0.92 to 3.85)	1.23 (0.45 to 3.39)	0.69 (0.28 to 1.68)	0.21** (0.05 to 0.80)
Facilitation_index	0.33*** (0.21 to 0.52)	0.18*** (0.06 to 0.55)	1.67* (0.98 to 2.86)	0.47 (0.14 to 1.58)
Precarity_index # Facilitation_index		4.83 (0.34 to 68.73)		37.89** (1.75 to 818.45)
Control_adult (ref: adult)	0.94 (0.72 to 1.22)	0.94 (0.72 to 1.22)	1.04 (0.76 to 1.42)	1.05 (0.76 to 1.43)
Control_sex (ref: male)	1.01 (0.80 to 1.29)	1.02 (0.80 to 1.29)	1.10 (0.82 to 1.49)	1.12 (0.83 to 1.52)
Control_distance	1.01 (0.85 to 1.18)	1.00 (0.85 to 1.18)	0.82* (0.67 to 1.02)	0.82* (0.66 to 1.02)
Duration_Symptoms (ref: <7 days)				
7–30 days	0.74** (0.57 to 0.95)	0.74** (0.57 to 0.95)	1.51*** (1.11 to 2.05)	1.50*** (1.11 to 2.04)
>30 days	0.50* (0.24 to 1.04)	0.51* (0.24 to 1.05)	1.22 (0.57 to 2.57)	1.25 (0.59 to 2.64)
Frequency (ref: no repeat illness)	1.14 (0.90 to 1.44)	1.14 (0.91 to 1.45)	0.93 (0.70 to 1.25)	0.94 (0.70 to 1.25)
Severity (ref: low)				
Moderate	0.61*** (0.48 to 0.79)	0.62*** (0.48 to 0.80)	1.00 (0.72 to 1.38)	1.02 (0.74 to 1.41)
Severe	0.64** (0.45 to 0.90)	0.64** (0.45 to 0.90)	1.04 (0.69 to 1.58)	1.06 (0.70 to 1.60)
Site (ref: Chiang Rai)	1.59 (0.69 to 3.66)	1.59 (0.69 to 3.67)	0.69 (0.24 to 1.98)	0.70 (0.24 to 2.01)
Constant	0.60 (0.23 to 1.57)	0.70 (0.26 to 1.89)	0.38 (0.11 to 1.26)	0.55 (0.16 to 1.91)
Observations	1421	1421	1421	1421
P value	<0.01	<0.01	<0.01	<0.01
AIC	1758.07	1758.72	1282.86	1279.49

Source: authors, based on survey data.

Illness-level data, including only completed illnesses experienced by respondent or a child under their supervision. Unweighted statistics. ORs adjusted by all covariates. 95% CIs in brackets.

*p<0.10, **p<0.05, ***p<0.01.

AIC, Akaike Information Criterion.

**Figure 2:**
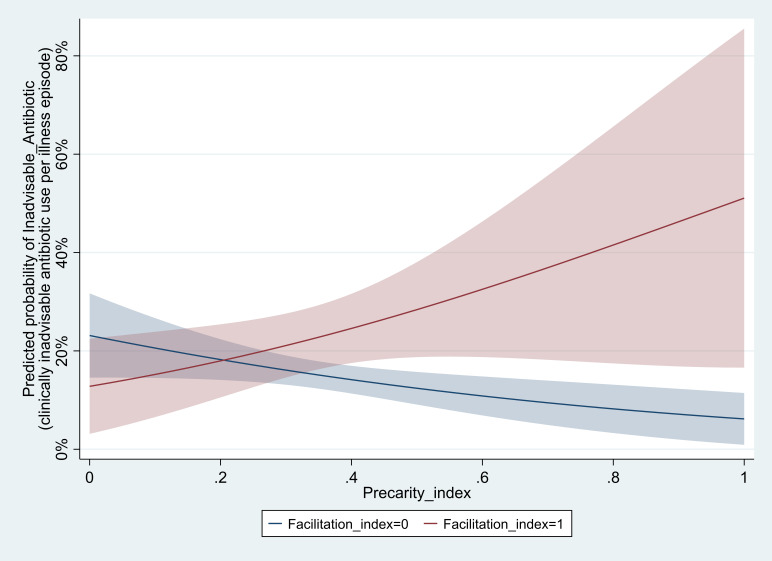
Predicted moderation effect of facilitation on the relationship between precarity index and clinically inadvisable antibiotic use. Source: authors, based on survey data. Notes: predicted and interpolated results on basis of model 4 in table 3, controlling for marginalisation, clinical determinants, field site and other control variables. Shaded areas indicate 95% CIs.

Figure 2 summarises the key findings from this analysis and illustrates that, in the absence of situational facilitators, higher degrees of precarity were predicted to link to more appropriate antibiotic access patterns (from a clinical perspective; indicating lower overuse of antibiotics). Simultaneously, patients exhibiting facilitation through mobile phones, motor transport or human support were predicted to be at higher risk of clinically inadvisable antibiotic use when being in otherwise precarious circumstances. For the highest levels of the precarity, the predicted difference between patients with and without facilitation in inadvisable antibiotic use was 44.9% points. In contrast, patients with a precarity index of zero were 10.3% points less likely to misuse antibiotic if they experienced the full range of facilitators captured in our survey.

## Discussion

Our research focused on the clinical and socioeconomic determinants of healthcare-seeking behaviour. We drew on a uniquely detailed health behaviour census survey from five villages across northern Thailand (Chiang Rai province) and southern Lao PDR (Salavan province) as part of a broader healthcare-seeking behaviour research project.^35^ Data from 2066 villagers across five communities included 1421 completed episodes of acute illnesses and injuries over the period of 6 months. Our four-step analysis offered the following interpretations:

1. Patients commonly exhibited respiratory tract symptoms, presence of an infection and fever. Precarious livelihoods were common in both sites, whereas indication of marginalisation was less widespread and more concentrated in Chiang Rai.
2. The common cold was positively associated with healthcare access whereas presence of an infection and respiratory tract symptoms were positively associated with antibiotic use. The surprisingly minor link between clinical presentation and antibiotic use pointed towards the importance of other, social determinants of healthcare-seeking behaviour, such as precarity and marginalisation.
3. We analysed the contribution of marginalisation and precarity to healthcare-seeking behaviour and found that situational facilitators were associated positively with healthcare access and antibiotic use, but no moderating effect of facilitation on precarity materialised. Marginalisation was negatively associated with antibiotic use. However, overall rates of healthcare access and antibiotic use could mask clinical justifications for the observed behaviour.
4. An evaluation of clinically inadvisable healthcare access and antibiotic use showed that facilitation independently linked to more appropriate healthcare access. In addition, controlling for other determinants of health behaviour, patients in otherwise precarious circumstances were significantly more likely to misuse antibiotics in the presence of situational facilitators.

To the best of our knowledge, this is the first study that examined quantitatively the relationship between precarity and AMR-related healthcare-seeking behaviour. A key strength of our study is the original micro-level behavioural data set, which enabled us to disentangle clinical presentation, marginalisation and precarity as separate drivers of healthcare-seeking behaviour.

Key limitations in this study pertain to the nature of the study sample, which reflects living conditions in selected rural communities of Chiang Rai and Lao PDR rather than broader rural populations of low-income and middle-income countries. More extensive and systematic health behaviour surveys based on our in-depth survey instrument would be necessary to understand the broader variability of common healthcare-seeking behaviours and their socioeconomic drivers. In addition, observational survey data on healthcare-seeking behaviour are subject to recall biases that could amplify the perspectives of relatively more privileged groups.^75^ However, considering complex behaviours in pluralistic health systems,^76–78^ community-level studies of this kind are essential to complement AMR research in clinical settings. Lastly, although our indicators of patients’ precarity and marginalisation were literature based, more extensive ethnographic research is necessary to identify their locally specific expressions, and to map out their links to healthcare-seeking behaviour and antimicrobial use.

## Conclusions

The increasing health policy interest in the 2030 Sustainable Development Agenda^79^ and its relationship to global health priority issues such as AMR^6^ underlines the importance of the social determinants of health. We therefore studied precarity and marginalisation alongside clinical indication as determinants of healthcare access and antibiotic use, using fine-grained health behaviour survey data from five rural communities.

Our study has two main implications. On the one hand, global health interventions must move beyond patient-centric and disease-centric approaches, acknowledging and responding to contextual factors that shape how people cope with illness. Interventions based on this logic could for instance aim at improving work environments, supporting social support structures and fostering community building. On the other hand, our study emphasises that localised forms of hardship could influence the effectiveness of clinical interventions to tackle AMR—an issue that is often overlooked in global health practice.^80–82^ Public health research and policy therefore require urgent collaboration with low- and middle-income country researchers and social scientists to appreciate the contextual drivers of global health challenges such as AMR.
