# Supplementary material for: Precarity and clinical determinants of healthcare-seeking behaviour and antibiotic use in rural Laos and Thailand
Source: BMJ Glob Health. 2020 Dec 9;5(12):e003779. doi: 10.1136/bmjgh-2020-003779 (PMC7733127; doi:10.1136/bmjgh-2020-003779)
Supplement: Supplementary data [file bmjgh-2020-003779supp001.pdf]

PRECARITY AND CLINICAL DETERMINANTS OF HEALTHCARE-SEEKING BEHAVIOUR AND ANTIBIOTIC USE IN RURAL LAOS AND THAILAND

Supplemental Material

Contents

VARIABLE CONSTRUCTION 1
STATISTICAL MODELS 2
SUPPLEMENTAL RESULTS 4
REFERENCES 6

Variable Construction

Supplemental Table 1. Clinical presentations according to symptoms declared by participants.

Clinical presentation	Symptoms declared
Fever	"Fever" as cited by the participant. Including, for example (in Thai / Lao): "ไข้" / "ໄຂ້" (*kai*, literally "fever") or "ตัวร้อน" / "ໂຕຮ້ອນ" (*tua ron* / *to hon*, literally "hot body").
Suspicion of an infection	Common cold, chills, "flu", sore throat, peritonsillar abscess, inflamed lungs, pneumonia, fever, Chickenpox, dengue virus, malaria, tetanus, cholera, leptospirosis, scrub typhus, fungal infection, Hand-Foot-Mouth disease, herpes infection, dysentery, appendicitis, vaginal discharge and urinary tract infection including cystitis.
Neurological presentation	Convulsion, headache, migraine and nerve pain
Respiratory presentation	Cough with or without blood, runny nose, "flu" as cited by the participant, sore throat, peritonsillar abscess, asthma, emphysema, pneumonia, and "inflamed lungs". Common cold was either cited by the participant or "flu" or by the presence of runny nose, sneezing or congestion without other complaint.
Digestive presentation	Stomach-ache, vomiting, gastritis, acidic reflux disease, diarrhoea, constipation, dysentery, appendicitis, Cholera, Beriberi, bloody stool and fatty liver.
Uro-gynecological presentation	Cystitis, kidney stones, inflamed kidney or uterus, vaginal discharge, menstruation, , miscarriage and mammary duct ectasia
Traumatism	Car or motorbike accident, broken leg or arm or wrist, torn ligament, skin abrasion or cut, pain from surgery, toothache, torn nail, falls from height, snake bite, back or knee pain, nerve pain, eye traumatism, herniated disc and tendinitis.

Source: Authors, based on survey data.
Notes: Coded based on free-text survey responses relating to symptom description and clinical diagnosis (if available).

Statistical Models

The corresponding logistic regression model for the Step 2 analysis estimated the probability of outcome *y* in illness episode *i* as a function of the respective symptom *s*, a matrix of other clinical determinants ***c***, precarity index *p*, facilitation index *f*, marginalisation index *m*, and a matrix of covariates ***x*** containing the remaining control variables:

$$\text{logit}[(y_\text{i} = 1 \mid s_i, p_i, f_i, m_i, \boldsymbol{c}_i, \boldsymbol{x}_i)] = \beta_1 + \beta_2 s_i + \beta_3 p_i + \beta_4 f_i + \beta_5 m_i + \boldsymbol{\beta c}_i + \boldsymbol{\beta x}_i, \quad (1)$$

where the probability of success P(y = 1) is expressed the natural log of the odds of achieving a positive result (conditional on x_i and z_i) and β and $\boldsymbol{\beta}$ (vector) are parameters [1]. Although not the focal point of analysis in Step 2, we also estimated the models with an interaction effect between precarity index *p* and facilitation index *f* to consider the potential moderating effect of the situational facilitators to ensure a consistent approach across Steps 2 to 4 and in light of goodness-of-fit of the regression models. The corresponding interaction model, where $p \times f_i$ denotes the interaction between the precarity and facilitation indexes, was defined as follows:

$$\text{logit}[(y_\text{i} = 1 \mid s_i, p_i, f_i, m_i, \boldsymbol{c}_i, \boldsymbol{x}_i)] = \beta_1 + \beta_2 s_i + \beta_3 p_i + \beta_4 f_i + \beta_5 p \times f_i + \beta_6 m_i + \boldsymbol{\beta c}_i + \boldsymbol{\beta x}_i, \quad (2)$$

For Step 3, we considered a reduced form of the model, excluding specific symptoms. We assessed the relative contribution of precarity, facilitation, and marginalisation to the outcomes of healthcare access and antibiotic use, based on their respective parameter estimates in the non-interacted and interacted models and controlling for other clinical determinants ***c*** and control variables ***x*** (age, sex, distance, site):

$$\text{logit}[(y_\text{i} = 1 \mid p_i, f_i, m_i, \boldsymbol{c}_i, \boldsymbol{x}_i)] = \beta_1 + \beta_2 p_i + \beta_3 f_i + \beta_4 m_i + \boldsymbol{\beta c}_i + \boldsymbol{\beta x}_i, \text{ and} \quad (3)$$

$$\text{logit}[(y_\text{i} = 1 \mid p_i, f_i, m_i, \boldsymbol{c}_i, \boldsymbol{x}_i)] = \beta_1 + \beta_2 p_i + \beta_3 f_i + \beta_4 p{\times}f_i + \beta_5 m_i + \boldsymbol{\beta c}_i + \boldsymbol{\beta x}_i, \quad (4)$$

In Step 4, we estimated again Models 3 and 4 depending on significance of the interaction term and goodness-of-fit, focusing in this final step of the analysis on the evaluative outcomes "Inadvisable_Antibiotic" and "Inadvisable_Access" to understand whether precarity in isolation or moderated by situational facilitators contributes to healthcare-seeking behaviour that appears clinically less desirable.

Supplemental Results

Supplemental Table 2. Variable description and summary statistics.

Variable name	*n*	Unique obs.	Mean	Std. dev.	Min.	Max.	Variable description
**Outcome variables**							
Access	1421	2	0.79	0.41	0	1	Binary variable: Patient accessed any kind of in-/formal care (1 = yes)
Antibiotic	1421	2	0.29	0.45	0	1	Binary variable: Antibiotic use during illness (1 = yes)
Inadvisable_Access	1421	2	0.34	0.47	0	1	Binary variable: Formal healthcare access without indication or vice versa
Inadvisable_Antibiotic	1421	2	0.17	0.38	0	1	Binary variable: Antibiotic use without indication or vice versa, or antibiotic use from informal sources
**Clinical presentation**							
Sepsis[a]	1421	2	0.65	0.48	0	1	Binary variable: Presence of an infection
Respi[a]	1421	2	0.65	0.48	0	1	Binary variable: Respiratory presentation (incl. common cold)
Common_Cold[a]	1421	2	0.37	0.48	0	1	Binary variable: Common cold
Fever[a]	1421	2	0.26	0.44	0	1	Binary variable: Fever
Neuro[a]	1421	2	0.19	0.39	0	1	Binary variable: Neurological presentation
Digest[a]	1421	2	0.15	0.36	0	1	Binary variable: Digestive presentation
Uro_Gyneco[a]	1421	2	0.02	0.13	0	1	Binary variable: Uro-gynaecological presentation
Trauma_Pain[a]	1421	2	0.13	0.33	0	1	Binary variable: Traumatism
Other[a]	1421	2	0.05	0.21	0	1	Binary variable: Other symptoms
Duration_Symptoms (categorical variable)							
<7 days	1421	2	0.63	0.48	0	1	Duration of symptoms: <7 days
7-30 days	1421	2	0.33	0.47	0	1	Duration of symptoms: 7-30 days
>30 days	1421	2	0.04	0.19	0	1	Duration of symptoms: >30 days
Severity (categorical variable)							
Low	1421	2	0.38	0.49	0	1	Symptom severity grade: low
Moderate	1421	2	0.43	0.49	0	1	Symptom severity grade: moderate
Severe	1421	2	0.19	0.39	0	1	Symptom severity grade: severe
Frequency	1421	2	0.41	0.49	0	1	Binary variable: Repeated illness of patient within six months (1 = yes)
**Other independent variables**							
Site	1421	2	0.56	0.50	0	1	Binary variable: Site (0= Chiang Rai, 1 = Salavan)
Precarity_index[b]	1421	7	0.36	0.17	0	1	Discrete variable: Precarity index (composed of 6 individual indicators)
Marginalisation_index[c]	1421	4	0.28	0.36	0	1	Discrete variable: Marginalisation index (composed of 3 individual indicators)
Facilitation_index[d]	1421	4	0.31	0.28	0	1	Discrete variable: Facilitation index (composed of 3 individual indicators)
Control_adult	1421	2	0.30	0.46	0	1	Binary variable: Illness of adult or child (0 = adult, 1 = <18 years)
Control_sex	1421	2	0.61	0.49	0	1	Binary variable: Sex of respondent (1 = female)
Control_distance	1421	783	2.91	2.86	0.01	7.32	Continuous variable: Distance to nearest formal healthcare provider (km)

Source: Authors, based on survey data.

Note: The correlation between three indexes ranged from 0% (precarity–facilitation) to +28% (precarity–marginalisation).

[a.] See Supplemental File 1 for variable construction.

[b.] Comprising 3 dimensions (6 indicators): occupational (non-contractual work, inflexible occupation), social (no adults in household, lack of health-related social contacts), and logistical (no household mobile phone, no motor transport).

[c.] Comprising 3 dimensions: education (no formal education), wealth (bottom quintile of provincial rural household assets), ethnicity (not belonging to majority ethnic group).

[d.] Comprising 3 dimensions: any illness-related social support, phone use, motor transport use (all during illness episode).

Supplemental Table 3. Crude and adjusted odds ratios for healthcare access according to clinical determinants in Chiang Rai, northern Thailand, and Salavan, southern Lao PDR.

Clinical determinants *	Healthcare access n (%)	OR (95% CI)	aOR (95% CI) **
Symptom severity grade			
- Low (n=699)	438 (62.7)	2.39 (1.93-2.95)	2.13 (1.80-2.53)
- Moderate (n=807)	659 (81.7)		
- High (n=381)	338 (88.7)		
Symptom duration			
- Less than a week (n=1,164)	821 (70.5)	1.56 (1.19-2.05)	1.90 (1.52-2.37)
- Between 1-4 weeks (n=619)	533 (96.1)		
- Over 4 weeks (n=104)	81 (77.9)		
Symptom frequency over the past 2 months[a]			
- Once (n=1,075)	839 (78.1)	0.89 (0.72-1.11)	0.95 (0.79-1.43)
- Twice (n=688)	502 (73.0)		
- More than twice (n=124)	94 (75.8)		
Clinical presentation			
- Fever[b] (n=365)	306 (83.8)	1.39 (0.95-2.05)	0.81 (0.55-1.20)
- Presence of an infection[c] (n=918)	757 (82.5)	1.38 (0.90-2.13)	1.32 (0.96-1.82)
- Neurological[d] (n=264)	202 (76.5)	0.97 (0.69-1.37)	0.89 (0.60-1.31)
- Respiratory overall[e] (n=925)	757 (81.8)	1.54 (1.06-2.23)	1.28 (0.93-1.77)
o Common cold[f] (n=519)	432 (83.2)	1.18 (0.81-1.73)	1.63 (1.17-2.26)
- Digestive[g] (n=215)	180 (83.7)	2.00 (1.31-3.07)	1.23 (0.77-1.98)
- Uro-gynecological[h] (n=23)	22 (95.7)	8.53 (1.13-64.65)	2.07 (0.26-16.74)
- Traumatism[i] (n=181)	140 (77.4)	1.76 (1.27-2.44)	0.92 (0.58-1.45)

Source: Authors, based on survey data.

Notes: Odds ratio adjusted by precarity, marginalisation, facilitation, duration, frequency and severity of symptoms, gender, age category, distance to the nearest formal healthcare, and by cluster, using a site-fixed control variable, with 95% confidence interval.

[a.] Symptom frequency was assessed on a two-month period

[b.] Fever as the main symptom named by the participant.

[c.] Presence of an infection included common cold, chills, “flu”, sore throat, peritonsillar abscess, inflamed lungs, pneumonia, fever, Chickenpox, dengue virus, malaria, tetanus, cholera, leptospirosis, scrub typhus, fungal infection, Hand-Foot-Mouth disease, herpes infection, dysentery, appendicitis, vaginal discharge, and urinary tract infection including cystitis.

[d.] Neurological presentation included convulsion, headache, migraine, and nerve pain.

[e.] Respiratory presentation overall included cough, cough with blood, runny nose, “flu” as named by the participant, sore throat, peritonsillar abscess, asthma, emphysema, pneumonia, and “inflamed lungs.”

[f.] Common cold was either cited by the participant or “flu” or by the presence of runny nose, sneezing or congestion without other complaint.

[g.] Digestive presentation included stomach-ache, vomiting, gastritis, acidic reflux disease, diarrhoea, constipation, dysentery, appendicitis, Cholera, Beriberi, bloody stool, and fatty liver.

[h.] Uro-gynaecological presentation included urinary tract infection including cystitis, kidney stones, inflamed kidney or uterus, vaginal discharge, menstruation, miscarriage, and mammary duct ectasia.

[i.] Traumatism included car or motorbike accident, broken leg or arm or wrist, torn ligament, skin abrasion or cut, pain from surgery, toothache, torn nail, falls from height, snake bite, back or knee pain, nerve pain, eye traumatism, herniated disc, and tendinitis.

Supplemental Table 4. Crude and adjusted odds ratios for antibiotic intake according to clinical determinants in Chiang Rai, northern Thailand, and Salavan, southern Lao PDR.

**Clinical determinants** [*]	**Antibiotic intake n (%)**	**OR (95% CI)**	**aOR (95% CI)** [**]
Symptom severity grade			
- Low (n=699)	156 (22.3)		
- Moderate (n=807)	236 (29.2)	1.34 (1.14-1.57)	1.28 (1.11-1.49)
- High (n=381)	128 (33.6)		
Symptom duration			
- Less than a week (n=1,164)	287 (24.7)		
- Between 1-4 weeks (n=619)	209 (33.8)	1.14 (0.92-1.40)	1.35 (1.13-1.63)
- Over 4 weeks (n=104)	24 (23.1)		
Symptom frequency over the past 2 months			
- Once (n=1,075)	305 (28.4)		
- Twice (n=688)	170 (24.7)	1.02 (0.84-1.23)	1.17 (0.99-1.38)
- More than twice (n=124)	45 (36.3)		
Clinical presentation			
- Fever[b] (n=365)	120 (32.9)	1.10 (0.81-1.49)	0.98 (0.74-1.32)
- Presence of an infection[c] (n=918)	298 (32.5)	1.57 (1.07-2.30)	1.44 (1.09-1.91)
- Neurological[d] (n=264)	70 (26.5)	0.99 (0.72-1.36)	0.87 (0.62-1.22)
- Respiratory overall[e] (n=925)	306 (33.1)	2.33 (1.64-3.31)	1.64 (1.23-2.18)
o Common cold[f] (n=519)	158 (30.4)	0.67 (0.49-0.91)	1.15 (0.89-1.48)
- Digestive[g] (n=215)	49 (22.8)	0.91 (0.63-1.31)	0.60 (0.41-0.87)
- Uro-gynecological[h] (n=23)	10 (43.5)	3.40 (1.42-8.12)	1.52 (0.61-3.77)
- Traumatism[i] (n=181)	55 (30.4)	1.89 (1.25-2.83)	1.32 (0.90-1.92)

Source: Authors, based on survey data.
Notes: Odds ratio adjusted by precarity, marginalisation, facilitation, duration, frequency and severity of symptoms, gender, age category, distance to the nearest formal healthcare, and by cluster, using a site-fixed control variable, with 95% confidence interval.
[a.] Symptom frequency was assessed on a two-month period
[b.] Fever as the main symptom named by the participant.
[c.] Presence of an infection included common cold, chills, "flu", sore throat, peritonsillar abscess, inflamed lungs, pneumonia, fever, Chickenpox, dengue virus, malaria, tetanus, cholera, leptospirosis, scrub typhus, fungal infection, Hand-Foot-Mouth disease, herpes infection, dysentery, appendicitis, vaginal discharge, and urinary tract infection including cystitis.
[d.] Neurological presentation included convulsion, headache, migraine, and nerve pain.
[e.] Respiratory presentation overall included cough, cough with blood, runny nose, "flu" as named by the participant, sore throat, peritonsillar abscess, asthma, emphysema, pneumonia, and "inflamed lungs."
[f.] Common cold was either cited by the participant or "flu" or by the presence of runny nose, sneezing or congestion without other complaint.
[g.] Digestive presentation included stomach-ache, vomiting, gastritis, acidic reflux disease, diarrhoea, constipation, dysentery, appendicitis, Cholera, Beriberi, bloody stool, and fatty liver.
[h.] Uro-gynaecological presentation included urinary tract infection including cystitis, kidney stones, inflamed kidney or uterus, vaginal discharge, menstruation, miscarriage, and mammary duct ectasia.
[i.] Traumatism included car or motorbike accident, broken leg or arm or wrist, torn ligament, skin abrasion or cut, pain from surgery, toothache, torn nail, falls from height, snake bite, back or knee pain, nerve pain, eye traumatism, herniated disc, and tendinitis.

References

1. Rabe-Hesketh S, Skrondal A: *Multilevel and longitudinal modeling using Stata: categorical responses, counts, and survival.* 3rd edn. College Station, TX: Stata Press; 2012.
